# MicroRNA-21 Regulates Diametrically Opposed Biological Functions of Regulatory T Cells

**DOI:** 10.3389/fimmu.2021.766757

**Published:** 2021-11-11

**Authors:** Jijun Sun, Ruiling Liu, Xiaozhen He, Jiang Bian, Wenbo Zhao, Weiyun Shi, Qingguo Ruan

**Affiliations:** ^1^ Eye Hospital of Shandong First Medical University, Jinan, China; ^2^ State Key Laboratory Cultivation Base, Shandong Provincial Key Laboratory of Ophthalmology, Shandong Institute of Ophthalmology, Shandong First Medical University & Shandong Academy of Medical Sciences, Qingdao, China; ^3^ Qingdao Eye Hospital of Shandong First Medical University, Qingdao, China

**Keywords:** miR-21, immune tolerance, immune-regulation, autoimmune disease, regulatory T cell

## Abstract

Regulatory T cells (Tregs) are considered important for controlling the onset and development of autoimmune disease. Although studies have shown that miR-21 is expressed at higher levels in Treg cells, it remains largely elusive whether miR-21 regulates the immune-suppressive function of Tregs. In the current study, we generated mice lacking miR-21 specifically in their Tregs and investigated the role of miR-21 in regulating Treg function both *in vitro* and *in vivo*. Our study revealed that Tregs lacking miR-21 exhibit normal phenotype and unaltered function in suppressing T cell proliferation and dendritic cell activation *in vitro*. However, compared with miR-21-sufficient Tregs, they produce significant more IL-17 and IL-10 when under pathogenic Th17-priming condition. Adenoviral delivery of miR-21 into Treg cells is able to reduce the expression of both IL-17 and IL-10. Mechanistic study revealed that miR-21 down-regulates IL-10 expression through direct targeting of IL-10, and suppresses reprogramming of Tregs into IL-17-secreting cells through down-regulating Stat3 activity. However, we detected no significant or marginal difference in the development of various autoimmune diseases between wild type mice and mice with Treg-specific deletion of miR-21. In conclusion, our study demonstrated that miR-21 in Tregs regulates diametrically opposed biological Treg functions and is largely dispensable for the development of autoimmune disease.

## Introduction

Regulatory T (Treg) cells are a specialized subpopulation of T cells that play crucial roles in maintaining immune homeostasis and preventing autoimmunity (1–3). Dysregulation in Treg cell frequency or function may lead to the development of autoimmune disease. The Foxp3 protein has generally been described as a master regulator of Treg cell development and function. Scurfy mice and patients carrying Foxp3 gene mutation display lymphoproliferation, lymphocytic infiltration, and multiorgan autoimmune diseases (4, 5). In addition, autoimmune diseases in the scurfy mice can be prevented by adoptive transfer of Treg cells (6). However, there is now substantial evidence that inflammatory conditions can cause the loss of Foxp3 expression and the generation of exTregs that adopt an effector phenotype (7–9). In addition, it has been shown that Tregs may display increased plasticity toward Th17 but retained suppressive function (10).

MicroRNAs (miRNAs) are small single-stranded non-coding RNA molecules that regulate gene expression by targeting mRNA for degradation or translational repression at the post-transcriptional level (11, 12). MicroRNA-21 (miR-21) is the most commonly up-regulated miRNA in a variety of cancers (13–15), but emerging evidence suggests that miR-21 up-regulation is also associated with the development of a variety of autoimmune diseases, such as psoriasis (16), experimental autoimmune encephalomyelitis (EAE) (17) and experimental autoimmune uveoretinitis (EAU) (18). Moreover, miR-21 knock-out mice are resistant to EAE (17) and lupus-like autoimmunity (19) and therapeutically targeting miR-21 has been shown to successfully treat various autoimmune diseases (17, 20–22). Further study demonstrated that the change in miR-21 expression was positively correlated with the expression of IL-17 in autoimmune disease. Indeed, miR-21 promotes Th17 differentiation by targeting and depleting SMAD-7, a negative regulator of TGF-β signaling (17). Alternatively, miR-21 can promote glucose metabolism of pathogenic Th17 cells by targeting the E3 ubiquitin ligase Peli1-c-Rel pathway (23).

Although miR-21 is expressed at higher levels in Tregs compared to conventional T cells (24), it is dispensable for iTreg differentiation *in vitro* and the development of thymic Treg (tTreg) and peripheral Treg (pTreg) *in vivo*, as reflected by unaltered Foxp3 expression and no developmental defect in the generation of tTreg and pTreg in the absence of miR-21 (17). However, other studies have shown that miR-21 acts as a positive regulator of Foxp3 expression, because increasing miR-21 level in non-Treg T cells or in naïve CD4^+^ T cells followed by polarization under Treg-priming condition significantly increases Foxp3 level (25, 26). In consistency with these results, it has been shown that miR-21 regulates the balance of Th17/Treg cells during T helper cell development in patients with rheumatoid arthritis (RA) by positively regulating Treg cell development (27). To make things even more complicated, it has been reported that treating Tregs *in vitro* with miR-21inhibitor significantly increases Foxp3 level and miR-21 negatively regulates the frequency of Treg cells and the expression of Foxp3 in peripheral blood mononuclear cell (28). On the other hand, whether miR-21 regulates the immune-suppressive function of Tregs remains largely elusive. The only evidence came from an *in vitro* study showing that Tregs treated with miR-21 inhibitor remain unchanged in their ability to suppress conventional T cell proliferation and dendritic cell activation (24).

To study the functional significance of Treg-specific expression of miR-21 under physiological conditions, we generated mice lacking miR-21 specifically in their Tregs and investigated the role of miR-21 in regulating Treg function both *in vitro* and *in vivo*. Our study revealed that, although Tregs lacking miR-21 produce significant more IL-17 and IL-10 when under pathogenic Th17-priming condition, miR-21-deficiency in Tregs is largely dispensable for the development of autoimmune disease.

## Materials and Methods

### Animals

MiR-21^flox/flox^ mice were on the C57BL/6 background and purchased from Shanghai Model Organisms Center, Inc. (Shanghai, China). Foxp3^Cre-YFP^ transgenic mice were kindly provided by Dr. Bin Li from Shanghai Jiao Tong University, P. R. China. Foxp3^Cre-YFP^ mice were crossed with miR-21^flox/flox^ mice to produce mice with Treg-specific deletion of miR-21 (Foxp3^Cre-YFP^miR-21^f/f^). 6 to 8-week old mice in the C57BL/6 background were purchased from Beijing Vital River Laboratory Animal Technology Company Limited. Mice were kept under pathogen-free conditions at the animal core facility of Shandong Eye Institute, Shandong First Medical University &Shandong Academy of Medical Sciences. All experiments were carried out in accordance with the Committee guidelines of Shandong Eye Institute and the Association for Research in Vision and Ophthalmology (ARVO) Statement for the Use of Animals in Ophthalmic and Vision Research.

### Induction and Clinical Evaluation of Autoimmune Disease in Mouse

For the induction of EAU in mouse, mice were anesthetized by intraperitoneal injection of pentobarbital sodium (80 mg/kg) and then immunized with 400 ug inter-photoreceptor retinoid-binding protein (IRBP)^1-20^ (5 mg/ml, GPTHLFQPSLVLDMAKVLLD, purchased from China Peptides) emulsified 1:1 in complete Freund’s adjuvant (Chondrex, USA) with an additional 100 µl mycobacterium tuberculosis H37R (5 mg/ml, BD biosciences, USA). An additional 200 ng bordetella pertussis toxin (Millipore, USA) was intravenously injected immediately after peptide injection. Fundoscopic exam was used to assess the severity of EAU on a scale of 0–4 using previously published criteria (29).

For the induction of EAE in mouse, 8 to 12-week old female mice were injected subcutaneously into both flanks with 100 μg myelin oligodendrocyte glycoprotein (MOG)^35–55^ peptide (3 mg/ml, MEVGWYRSPFSRVVHLYRNGK, purchased from China Peptides) dissolved in PBS and emulsified in an equal volume of complete Freund’s adjuvant (Chondrex, USA) supplemented with 5 mg/ml Mycobacterium tuberculosis H37R. Mice were also injected intraperitoneally (i.p.) with 200 ng pertussis toxin (Millipore, USA) on the day of immunization and 48 h later. Clinical assessment of EAE was performed daily after disease induction according to the following criteria: 0, no disease; 1, tail paralysis; 2, hind limb weakness or partial paralysis; 3, complete hind limb paralysis; 4, forelimb and hind limb paralysis; 5, moribund state. Mean clinical scores were calculated by adding the scores of individual mice and dividing by the total number of mice in each group.

For the induction of RA in mouse, 6 to 8-week old male mice were intradermally immunized with 100 μg chicken type II collagen (4 mg/ml, purchased from China Peptides) emulsified in complete Freund’s adjuvant at the base of the tail, followed by a booster immunization with 100 μg chicken type II collagen emulsified in incomplete Freund’s adjuvant. The cumulative score of all four paw scores were calculated on a scale of 0–16, where each paw was scored as follows: 0, normal paw; 1, one toe inflamed and swollen; 2, more than one toe, but not entire paw inflamed and swollen, or mild swelling of entire paw; 3, entire paw inflamed and swollen; 4, very inflamed and swollen or ankylosed paw.

### Antibodies and Flow Cytometry Analysis

Flow cytometry analyses were performed on freshly isolated cells from mouse thymus, spleen, and mesenteric lymph node (MLN) or cultured cells. Cells were labeled with a combination of the following fluorescence-conjugated mouse mAbs: PE or PerCP-Cy5.5-anti-CD4, APC or PerCP-Cy5.5-anti-CD8, PerCP-Cy5.5-anti-CD25, PE-cy7-anti-GITR, PerCP-Cy5.5-anti-Nrp1, PerCP-Cy5.5-anti-CD44, APC-anti-CD62L, PerCP-Cy5.5-anti-CD11c. For the intracellular staining of Foxp3, RORγT and CTLA4, cells were fixed, permeabilized, and stained with PE or APC-anti-Foxp3, PE-anti-RORγT and APC-anti-CTLA4 per manufacturer’s instructions (Invitrogen, USA). For the intracellular staining of IL-17A, IL-17F and IL-10, cells were stimulated with cell stimulation cocktail (PMA+Ionomycin) plus protein transport inhibitors (eBioscience, USA) for 4 h and then stained with APC-anti-IL-17A, PE or APC-anti-IL-17F, or APC-anti-IL-10 per manufacturer’s instructions (Invitrogen, USA). For the intracellular staining of phospho-Stat3, cells were fixed and permeabilized by transcription factor phospho buffer set, and then stained with Alexa Fluor 647-anti-Stat3 (PY705) antibody (BD Bioscience, USA) per manufacturer’s instructions (BD Bioscience, USA). Stained cells were examined on CytoFLEX flow cytometry system (Beckman Coulter Inc., USA). All antibodies were purchased from BioLegend.

### *In Vitro* Treg Suppression Assay

For the isolation of Treg cells (CD4^+^CD8^-^YFP^+^), CD4^+^ T cells from spleen were first enriched using EasySep Mouse CD4^+^ T Cell Isolation Kit (STEMCELL Technologies, Canada). Cells were then stained with PE-anti-CD4 and APC-anti-CD8, and CD4^+^CD8^-^YFP^+^ Treg cells were sorted by FACSAria III cell sorter (Becton Dickinson, USA). For *in vitro* Treg suppression of T cell proliferation, naïve CD4^+^ T cells were isolated from the spleen of WT mice using EasySep™ Mouse naïve CD4^+^ T Cell Isolation Kit (STEMCELL Technologies, Canada). Cells were further labeled with 4 μM eFluor-670 (Invitrogen, USA) for 10 min at 37°C followed by washing with ice-cold complete RPMI-1640 media. Sorted Treg cells were mixed with eFluor-670 labeled naïve CD4^+^ T cells (2.5×10^4^/well) at different ratios and seeded in 96-well plate. Mouse T-Activator CD3/CD28 beads (Gibco, USA) were added at a ratio of 1: 1 (beads: naïve CD4^+^ T cells). Dilution of eFluor-670 was analyzed by flow cytometry. For *in vitro* Treg suppression of dendritic cell activation, bone marrow-derived dendritic cells (BMDCs) were generated from femoral and tibial bones cells as previously described (30). BMDCs (0.5×10^5^/well) were either cultured alone or mixed with sorted Treg cells at a ratio of 1:1 and seeded in 96-well plate. Cells were treated with 2 ug/ml anti-CD3 (ebioscience, USA), 2 ug/ml anti-CD28 (ebioscience, USA), 100 IU/ml IL-2 (PeproTech, USA) and 10 ng/ml LPS (Sigma, USA). After co-culture for 12 h, cells were collected and stained with PerCP-Cy5.5-anti-CD11c, APC-anti-CD86 and APC-anti-CD80 according to manufacturer’s instructions (Invitrogen, USA), and analyzed by flow cytometry.

### Enzyme-Linked Immunosorbent Assay

For cytokine assays, flow sorted Treg cells (CD4^+^CD8^-^YFP^+^) from spleen, inguinal lymph node (ILN), or cervical lymph node (CLN) were cultured at a density of 0.2×10^6^/well (96-well plate) in 100 µl of complete RPMI-1640 culture medium (Corning, USA) and stimulated with 2 ug/ml anti-CD3 (ebioscience, USA), 2 ug/ml anti-CD28 (ebioscience, USA) and 100 IU/ml IL-2 (PeproTech, USA) in the presence or absence of 50 ng/ml IL-6 (PeproTech, USA), 20 ng/ml IL-1β (PeproTech, USA), 20 ng/ml IL-23 (PeproTech, USA) or 10 ng/ml IL-12 (PeproTech, USA). After 48 h, the concentrations of IL-17A, IL-17F, IFN-γ, IL-10 and TGF-β in the culture supernatants were determined by quantitative enzyme-linked immunosorbent assay (ELISA) per manufacturer’s instructions (Invitrogen, USA). For the preparation of tissue extract, mouse eye, brain or paw were homogenized in PBS containing complete protease inhibitor mixture (Roche, USA) using TissueLyser II per manufacturer’s instructions (QIAGEN, Germany). The concentrations of IL-17A, IL-17F, IFN-γ, and TNF-α in the tissue extract were determined by ELISA as described above.

### RNA Isolation and RT-PCR

Total RNA was isolated using TRIzol reagent following manufacturer’s instructions (Life Technologies, USA). RNA samples were reversely transcribed using the primescript reverse transcription kit (Takara, JPN). The expression of mouse miR-21, IL-17A, IL-17F, IFN-γ, IL-10, TGF-β, CCR-6, Ebi3, Gzmb and Prf1 was determined by quantitative RT-PCR using specific primers and the Applied Biosystems 7500 system. Relative levels of expression were determined using U6 (for miR-21) or GAPDH (for all others) as internal control. The primers for the reverse transcription and detection of miR-21 were supplied by RiboBio (Guangzhou, China). The primers for the detection of all other genes were synthesized by Invitrogen (USA). Primer sequences are as follows (forward and reverse): IL-17A, 5’-CTCCAGAAGGCCCTCAGACTAC-3’ and 5’-AGCTTTCCCTCCGCATTGACACAG-3’; IL-17F, 5’-CTGTTGATGTTGGGACTTGCC -3’ and 5’-TCACAGTGTTATCCTCCAGG -3’; IFN-γ, 5’-TTCTGGCTGTTACTGCCACGG-3’ and 5’-GCCTTGCTGTTGCTGAAGAAG-3’; IL-10, 5’-CTTACTGACTGGCATGAGGATCA-3’ and 5’- CCTGCATTAAGGAGTCGGTTAGC-3’; TGF-β, 5’- CTTCAATACGTCAGACATTCGG-3’ and 5’-AGCCACTCAGGCGTATCAGTG-3’; CCR-6, 5’-CCTGGGCAACATTATGGTGGT-3’ and 5’-CAGAACGGTAGGGTGAGGACA-3’; Prf1, 5’-AGCACAAGTTCGTGCCAGG-3’ and 5’-TGGCGTCTCTCATTAGGGAG-3’; Gzmb, 5’-CCACTCTCGACCCTACATGG-3’ and 5’-CCTTCACAGTGAGCAGCAGTC-3’; Ebi3, 5’-CTTACAGGCTCGGTGTGGC-3’ and 5’-GTGACATTTAGCATGTAGGGCA-3’; GAPDH, 5’-GGTCGGTGTGAACGGATTTGG-3’ and 5’-CCGTGAGTGGAGTCATACTGG-3’.

### Luciferase Assay

Mouse IL-10 3’-untranslated region (3’UTR) (+1 to +702) containing the miR-21-5p binding site was inserted into the NheI/XbaI site downstream of the luc2 gene of pmirGLO luciferase reporter plasmid (Promega, Madison, USA). Control and miR-21-5p agomir were purchased from Ruibo (Guangzhou, China). For dual-luciferase assay, CD4^+^CD8^-^YFP^+^ Treg cells from the spleen of WT and Foxp3^Cre-YFP^miR-21^f/f^ mouse were flow sorted and transfected with mouse IL-10 3’UTR reporter plasmid alone or in combination with miR-21-5p agomir using Nucleofector Kit for mouse T cells (Lonza, Switzerland) per manufacturer’s instructions. Transfected cells were then cultured in the presence of 2 μg/ml anti-CD3, 2 μg/ml anti-CD28, 500 IU/ml IL-2, and 10 ng/mL TGF-β. After 48 h, the luciferase activities of the whole-cell lysate were analyzed using a dual-luciferase reporter assay system (Promega, USA). Data were normalized for transfection efficiency by dividing firefly luciferase activity by that of the Renilla luciferase.

### *In Vivo* BrdU Assay

Mice were intraperitoneally injected with 100 µl of 10 mg/ml BrdU. Administration of BrdU was repeated every 12 h for three consecutive days. Total cells were isolated from thymus, spleen and MLN 12 h after the last treatment. Cell surface staining was first performed with PE-anti-CD4 and PerCP-Cy5.5-anti-CD8. Cells were then stained with APC-anti-BrdU according to manufacturer’s instructions (BD Bioscience, USA) and analyzed by flow cytometry.

### Fluorescent Dye-Based Cell Proliferation Assay

Treg cells (CD4^+^CD8^-^YFP^+^) were flow sorted as described above and incubated with 4 μM eFluor-670 (Invitrogen, USA) at 37°C for 15 min. The reaction was stopped with the same volume of cold RPMI-1640 containing 10% heat-inactivated FBS. After washing three times with PBS, eFluor670-labeled cells were cultured at 2×10^5^ cells/well (96-well plate) and treated with 100 IU/ml IL-2, 2 ug/ml anti-CD3 and 2 ug/ml anti-CD28 for 72 h. Cell proliferation was then analyzed by flow cytometry.

### Apoptosis Detection

Apoptosis detection were performed on freshly isolated Tregs or Tregs treated with α-CD3 (2 ug/ml) plus α-CD28 (2 ug/ml) in the presence of IL-2 (100 IU/ml), TNF-α (5 ng/ml), IL-1β (4 ng/ml) and IFN-γ (25 ng/ml) for 96 h. The percentage of apoptotic cells was determined by Annexin V/7AAD (7-Amino-Actinomycin D) staining according to manufacturer’s instructions (KeyGEN BioTECH, China). Briefly, Treg cells were stained with APC-anti-Annexin V and 7-AAD followed by analysis using flow cytometry (Beckman Coulter Inc, USA). Apoptotic cells were defined as Annexin V and 7-AAD double-positive cells.

### Adenovirus Infection

Adenovirus over-expressing miR-21 was designed and supplied by Vigene Biosciences, China. Because of the color conflict, Treg cells (CD4^+^CD25^+^) from the spleen of C57BL/6 mice were isolated using EasySep Mouse CD4^+^CD25^+^ Treg Cell Isolation Kit (STEMCELL Technologies, Canada). Isolated Treg cells were first cultured in complete RPMI-1640 medium at a density of 2×10^5^/well in 96-well plate and stimulated with 100 IU/ml IL-2 in the presence of 2 ug/ml anti-CD3 plus 2 ug/ml anti-CD28 for 36~48 h. Then 9x10^10^ pfu adenovirus was added to the plate. After 12 h, replace culture medium with fresh complete RPMI-1640 medium in the presence of 2 μg/mL anti-CD3, 2 μg/mL anti-CD28, 100 IU/mL IL-2, and continue to culture for 36 h. Transduced cells were then cultured in the presence of 50 ng/ml IL-6, 20 ng/ml IL-1β, 20 ng/ml IL-23 for 72 h. Transduction efficiency was ~60-70% as determined by examination under a fluorescent microscope.

### Statistical Analysis

The data were analyzed using GraphPad Prism 8.0 and expressed as mean ± SD. The student’s t-test was used to compare the significance of the difference between two groups. ANOVA was utilized for multi-group comparison, with Tukey’s test for subsequent post-hoc comparisons between two groups. Differences were considered statistically significant at *P<0.05, **P<0.01, and ***P<0.001.

## Results

### Treg Cells Lacking miR-21 Display Normal Phenotype

**Figure 1:**
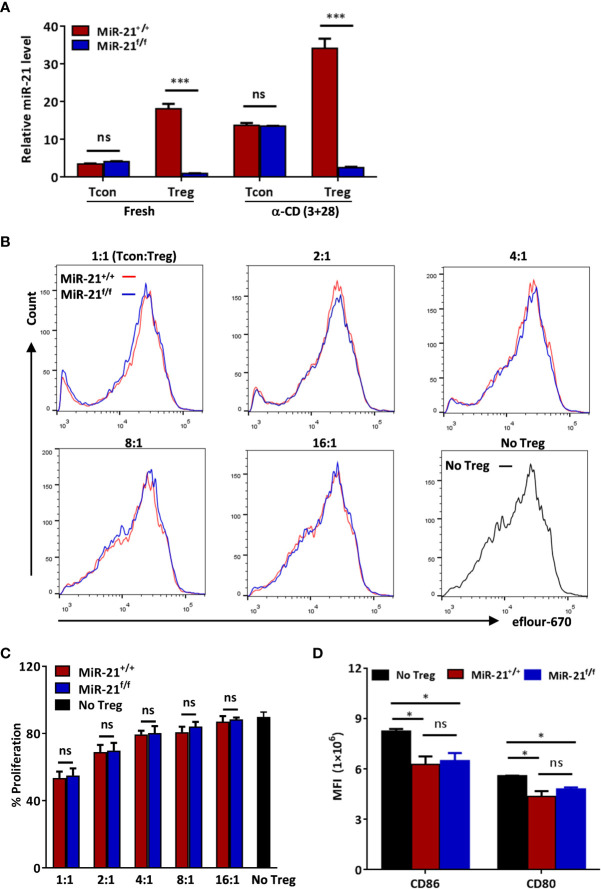
MiR-21 deficiency doesn’t affect the immune-suppressive function of Treg cells *in vitro*. **(A)** Deletion of miR-21 in both rested Treg (rTreg) and activated Treg (aTreg) was confirmed by RT-PCR. CD4^+^CD8^-^YFP^-^ (Tcon) and CD4^+^CD8^-^YFP^+^ (Treg) were flow sorted from the spleen of Foxp3^Cre-YFP^miR-21^+/+^ (MiR-21^+/+^) and Foxp3^Cre-YFP^miR-21^f/f^ (MiR-21^f/f^) mice (n=5). Cells were either untreated or treated with α-CD3 plus α-CD28 for 24 h, and miR-21 level was determined by quantitative RT-PCR. **(B, C)** Flow sorted splenic CD4^+^CD8^-^YFP^+^ cells from Foxp3^Cre-YFP^miR-21^+/+^ (MiR-21^+/+^) and Foxp3^Cre-YFP^miR-21^f/f^ (MiR-21^f/f^) mice (n=3) were mixed with eFluor-670-labeled naïve CD4^+^ T cells at indicated ratio and incubated with mouse T-Activator CD3/CD28 beads. After 72 h, dilution of eFluor-670 was examined by flow cytometry. The histogram **(B)** and quantification **(C)** of the proliferating T cells were shown. **(D)** BMDCs were cultured alone or mixed with flow sorted splenic CD4^+^CD8^-^YFP^+^ cells from either Foxp3^Cre-YFP^miR-21^+/+^ (MiR-21^+/+^) or Foxp3^Cre-YFP^miR-21^f/f^ (MiR-21^f/f^) mice (n=3) at 1:1 ratio. Cells were then treated with anti-CD3, anti-CD28, IL-2 and LPS for 12 h. The MFI of CD80 and CD86 in BMDCs was examined by flow cytometry after cells were stained with fluorescent-labeled antibodies against CD11c, CD80 and CD86. Results are representative of two to three independent experiments. MLN, mesenteric lymph node; BMDCs, bone-marrow derived dendritic cells; MFI, mean fluorescence intensity. ns, no significance. *p < 0.05; ***p < 0.001.

To study the functional significance of Treg-specific expression of miR-21 under physiological conditions, we generated mice with Treg specific deletion of miR-21 (Foxp3^Cre-YFP^miR-21^f/f^). Deletion of miR-21 in both rested Treg (rTreg) and activated Treg (aTreg) was confirmed by RT-PCR, while miR-21 expression in freshly isolated and activated conventional T cells was not affected (**Figure 1A**). Thymic and peripheral T cell development was normal in Foxp3^Cre-YFP^miR-21^f/f^ mice (**Figure S1A**). The proportions (**Figure S1A**) and numbers (**Figure S1B**) of CD4^+^CD8^-^YFP^+^ Treg cells from thymus, spleen and mesenteric lymph node (MLN) were comparable between Foxp3^Cre-YFP^miR-21^+/+^ (WT) and Foxp3^Cre-YFP^miR-21^f/f^ mice. We further confirmed normal development of Foxp3^Cre-YFP^miR-21^f/f^ tTreg cells based on proportional expression of Nrp-1 (**Figure S1C**). The phenotype of Treg cells, characterized by the expression Foxp3, CD25, cytotoxic T lymphocyte-associated protein 4 (CTLA-4) and glucocorticoid-induced TNFR-related protein (GITR), was also similar between WT and Foxp3^Cre-YFP^miR-21^f/f^ mice (**Figure S2A**). Furthermore, we observed a similar outcome for Treg cell phenotype when splenic Treg cells were activated and treated with pathogenic Th17-priming cytokines (IL-6+IL-1β+IL-23) or Th1-priming cytokine (IL-12) *in vitro* (**Figure S2B**). CD25 expression was not examined in this experimental setting because it is also a T cell activation marker.

Because it has been reported that miR-21 inhibits cell apoptosis and promotes cell proliferation, we then assessed the apoptosis and proliferation of Treg cells from WT and Foxp3^Cre-YFP^miR-21^f/f^ mice. As shown in **Figures S3A, B**, the apoptosis ratio, as determined by the proportion of Annexin V^+^7AAD^+^ cells, was similar in fresh splenic Tregs and Tregs treated with inflammatory cytokine mix (TNF-α+IL-1β+IFN-γ) *in vitro*. Next we examined the proliferation of Tregs *in vitro* using fluorescent dye-based cell proliferation assay. Our results showed that miR-21-deficiency in Treg cell does not affect the proliferation of Tregs when cultured *in vitro* with IL-2 in the absence or presence of activation (anti-CD3 plus anti-CD28) (**Figures S4A, B**). In addition, we used labeling with the thymidine analog BrdU to assess Treg cell proliferation *in vivo*. We found that the proportions of BrdU-positive Treg cells in the thymus, spleen and MLN were also similar between WT and Foxp3^Cre-YFP^miR-21^f/f^ mice (**Figures S4C, D**). Taking together, these results indicate that miR-21-deficiency in Treg cells doesn’t affect their development, phenotype, apoptosis and proliferation.

### Treg Cells Lacking miR-21 Exhibit Normal Immune-Suppressive Function

Because Treg cells play a key role in the control of pathological immune responses, we next sought to determine whether miR-21-deficiency affects the immune-suppressive function of Tregs. MiR-21-deficiency in Tregs does not cause any apparent global immune abnormalities as reflected by no splenomegaly or lymphadenopathy was observed in any of the Foxp3^Cre-YFP^miR-21^f/f^ mice (**Figure S5**). Fractions of splenic naïve and memory T cells in naïve mice were also comparable between WT and Foxp3^Cre-YFP^miR-21^f/f^ mice (**Figure S6**). Next, we examined the *in vitro* suppressive activity of Treg cells. MiR-21-deficient splenic Treg cells proved as effective as WT Treg cells in inhibiting T cell proliferation (**Figures 1B, C**). It has been reported that Treg cells can suppress T cell proliferation indirectly by down-regulating co-stimulatory molecules on antigen presenting cells (such as dendritic cells) (31). We then examined the expression of CD80 and CD86 on bone-marrow derived dendritic cells (BMDCs) co-cultured with splenic Tregs from either WT or Foxp3^Cre-YFP^miR-21^f/f^ mice. Our results showed that Tregs from WT and Foxp3^Cre-YFP^miR-21^f/f^ mice exhibit no significant difference in reducing the expression of CD80 and CD86 on BMDCs (**Figure 1D**).

### Treg Cells Lacking miR-21 Produce Significant More IL-17 and IL-10 *In Vitro*

**Figure 2:**
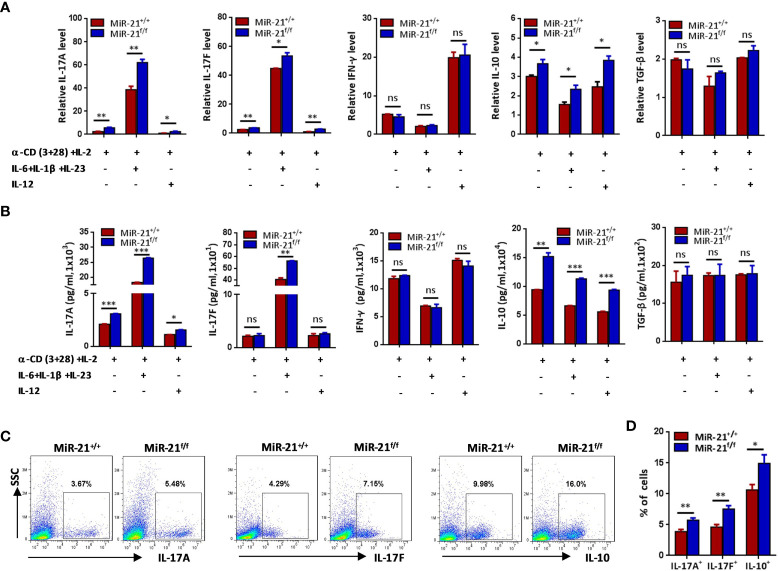
Treg cells lacking miR-21 produce significant more IL-17 and IL-10 *in vitro*. CD4^+^CD8^-^YFP^+^ cells were flow sorted from the spleen of Foxp3^Cre-YFP^miR-21^+/+^ (MiR-21^+/+^) and Foxp3^Cre-YFP^miR-21^f/f^ (MiR-21^f/f^) mice (n=5). **(A, B)** Cells were stimulated with anti-CD3 plus anti-CD28 in the presence of indicated Th0, Th1 or Th17 priming cytokines. 48 h after stimulation, total RNA was extracted, and the mRNA expression of IL-17A, IL-17F, IFN-γ, IL-10, and TGF-β was determined by quantitative RT-PCR **(A)**. In addition, culture supernatants were collected and the protein level of IL-17A, IL-17F, IFN-γ, IL-10, and TGF-β was determined by ELISA **(B)**. **(C, D)** Cells were stimulated with anti-CD3 plus anti-CD28 in the presence of Th17 priming cytokines. After 72 h, cells were treated with PMA plus ionomycin in the presence of GolgiStop for 4 h. Cells were then fixed, permeabilized, and stained with anti-Foxp3, anti–IL-17A, anti–IL-17F, anti–IL-10, and examined by flow cytometry **(C)**. The quantification of the percentages of IL-17A^+^, IL-17F^+^ and IL-10^+^ cells among total live cells were shown in **(D)**. Data are representative of three independent experiments. ns, no significance. *p < 0.05, **p < 0.01, ***p < 0.001.

**Figure 3:**
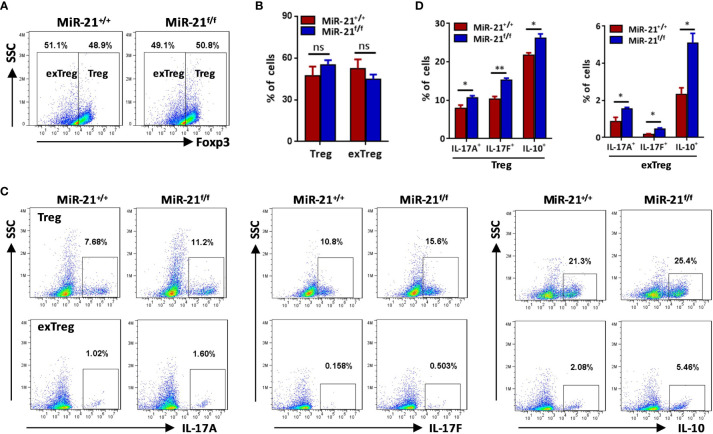
The proportions of IL-17^+^ and IL-10^+^ cells were significantly increased by both exTregs and Tregs that lacking miR-21. CD4^+^CD8^-^YFP^+^ cells were flow sorted from the spleen of Foxp3^Cre-YFP^miR-21^+/+^ (MiR-21^+/+^) and Foxp3^Cre-YFP^miR-21^f/f^ (MiR-21^f/f^) mice (n=5). Cells were stimulated with anti-CD3 plus anti-CD28 in the presence of Th17 priming cytokines. After 72 h, cells were treated with PMA plus ionomycin in the presence of GolgiStop for 4 h. Cells were then fixed, permeabilized, and stained with anti-Foxp3, anti–IL-17A, anti–IL-17F, anti–IL-10, and examined by flow cytometry. The proportions of Foxp3^+^ (Treg) and Foxp3^-^ (exTreg) cells among total live cells were shown in **(A, B)**. In addition, the proportions of IL-17A^+^, IL-17F^+^ and IL-10^+^ cells among Foxp3^+^ (Treg) and Foxp3^-^ (exTreg) cells were also examined by flow cytometry **(C)** and the quantification of those results was shown in **(D)**. Data are representative of three separate experiments. ns, no significance. *p < 0.05, **p < 0.01.

Secreting inhibitory cytokines, such as IL-10 and TGF-β, is one of the many effector pathways utilized by Treg cells to control immune response. On the other hand, inflammatory environments can cause the loss of Foxp3 expression and the generation of exTregs that gain an effector phenotype. Therefore, we examined re-programming of miR-21-deficient splenic Treg cells treated with anti-CD3 plus anti-CD28 in the presence of Th0, Th1 or pathogenic Th17 priming cytokines. Our results showed that under pathogenic Th17 priming condition, the levels of IL-17 mRNA and protein were significantly increased, while under Th1 priming condition, the levels of IFN-γ mRNA and protein were significantly increased (**Figures 2A, B**). Furthermore, we found that IFN-γ expression was comparable between WT and miR-21-deficient Treg cells upon above-mentioned destabilizing treatments. By contrast, the mRNA expression (**Figure 2A**) and secretion of IL-17A and IL-17F (**Figure 2B**) by miR-21-deficient Treg cells were significantly increased compared with WT Treg cells. In addition, we found that the mRNA and protein levels of IL-10, but not TGF-β, were significantly increased by miR-21-deficient Treg cells (**Figures 2A, B**). We further confirmed the up-regulation of IL-17 and IL-10 by miR-21-deficient Treg cells under pathogenic Th17-priming condition using flow cytometry (**Figures 2C, D**). However, Foxp3 was down-regulated to similar degrees in WT and miR-21-deficient Treg cells, as the proportion of exTregs (lost Foxp3 expression) and Tregs (maintained Foxp3 expression) were comparable between WT and miR-21-deficient Treg cells (**Figures 3A, B**). We further showed that the proportion of IL-17^+^ and IL-10^+^ cells were significantly increased by both exTregs and Tregs that lacking miR-21 (**Figures 3C, D**). It shall be noted that although ~50% of Tregs lost Foxp3 expression when cultured *in vitro* under Th17-inducing condition, these exTregs may not be the same as exTregs generated *in vivo*. For example, *in vitro* generated exTregs actually produce reduced IL-17 as shown in **Figure 3C**. We examined the expression of RORγT and found that, although *in vitro* generated exTregs lost Foxp3 expression, they exhibited reduced RORγT expression when compared with Tregs that retained Foxp3 expression (**Figure S7**). Although this result is consistent with that of reduced IL-17 expression by exTregs generated *in vitro*, it remains elusive how RORγT expression was down-regulated in those Tregs.

### MiR-21 in Treg Cells Inhibits Il-17 Expression by Down-Regulating Stat3 Activity and Reduces IL-10 Expression Through Direct Targeting of IL-10

**Figure 4:**
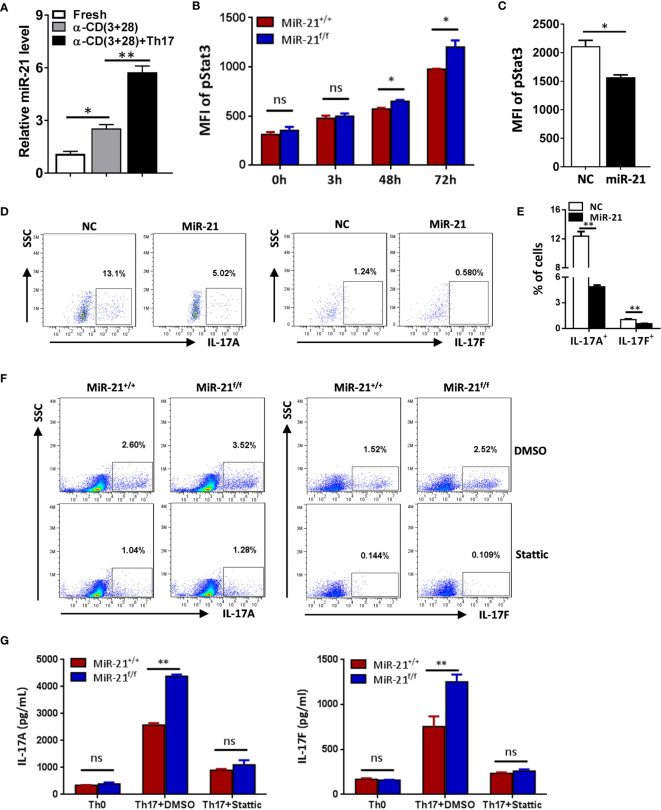
MiR-21 in Treg cells suppresses IL-17 expression through down-regulating Stat3 activity. **(A)** CD4^+^CD8^-^YFP^+^ cells were flow sorted from the spleen of Foxp3^Cre-YFP^miR-21^+/+^ mice (n=3) and either untreated or treated with α-CD3 plus α-CD28 in the absence or presence of Th17 priming cytokines for 48 h, and then miR-21 level was determined by quantitative RT-PCR. **(B)** CD4^+^CD8^-^YFP^+^ cells were flow sorted from the spleen of Foxp3^Cre-YFP^miR-21^+/+^ (MiR-21^+/+^) and Foxp3^Cre-YFP^miR-21^f/f^ (MiR-21^f/f^) mice (n=5). Cells were then cultured with α-CD3 plus α-CD28 in the presence Th17 priming cytokines. Cells were collected at the indicated time-points and fixed, permeabilized, and stained with anti-Stat3. The MFI of pStat3 was determined by flow cytometry. **(C-E)** CD4^+^CD25^+^ Treg cells were isolated from the spleen of C57BL/6 mice using EasySep Mouse CD4^+^CD25^+^ Treg Cell Isolation Kit and cultured with α-CD3 plus α-CD28 and IL-2 for 48 h before control adenovirus (NC) or adenovirus over-expressing miR-21 (miR-21) were added. After culture for another 48 h, cells were treated with Th17 priming cytokines for 72 h. Cells were then fixed, permeabilized, stained with anti-Stat3, anti–IL-17A, anti–IL-17F, and examined by flow cytometry. The quantification of the MFI of pStat3 was shown in **(C)**. The percentages of IL-17A^+^ and IL-17F^+^ cells were plotted **(D)** and quantified **(E)**. Cells shown in **(D)** were gated on transduced cells. **(F, G)** CD4^+^CD8^-^YFP^+^ cells were flow sorted from the spleen of Foxp3^Cre-YFP^miR-21^+/+^ (MiR-21^+/+^) and Foxp3^Cre-YFP^miR-21^f/f^ (MiR-21^f/f^) mice (n=5). Cells were pre-treated with either DMSO or Stat3 inhibitor (Stattic) for 1h and then cultured with α-CD3 plus α-CD28 in the presence of Th17 priming cytokines for 72 h. Cells were then fixed, permeabilized, and stained with anti–IL-17A, anti–IL-17F, and examined by flow cytometry **(F)**. In addition, culture supernatants were collected and the protein levels of IL-17A and IL-17F **(G)** were determined by ELISA. Cells cultured with α-CD3 plus α-CD28 (Th0) were used as negative control for Th17 cell differentiation. Data are representative of two to three separate experiments. ns, no significance. *p < 0.05, **p < 0.01.

To determine how miR-21 in Treg cells inhibits IL-17 expression, we first examined miR-21 expression in both freshly isolated and activated Treg cells in the absence or presence of pathogenic Th17-priming cytokines. Our results showed that while miR-21 expression in Treg cells was increased upon activation, it is further increased when Treg cells were activated in the presence pathogenic Th17-priming cytokines (**Figure 4A**). This is not surprising because pathogenic Th17-priming cytokines including IL-6, IL-1β and IL-23 can all activate Stat3, an important transcription factor that promotes miR-21 expression. Surprisingly, while phosphor-Stat3 (pStat3) level was increased in Treg cells upon treatment with pathogenic Th17-priming cytokines, miR-21-deficient Treg cells exhibit significantly elevated pStat3 level compared with WT Treg cells (**Figure 4B**). These results indicate that although Stat3 in Treg cells promotes miR-21 expression, miR-21 is able to down-regulate Stat3 activity in a negative feedback manner. We further confirmed this hypothesis by showing that adenoviral delivery of miR-21 into Treg cells is able to reduce pStat3 level (**Figure 4C**) and the proportion of IL-17^+^ cells (**Figures 4D, E**). To determine whether miR-21 reduces the proportion of IL-17^+^ cells through down-regulating Stat3 activity, we treated Treg cells with Stat3 inhibitor (Stattic) and then examined the proportion of IL-17^+^ cells upon treatment with pathogenic Th17-priming cytokines. Our results showed that the proportion of IL-17^+^ cells is no longer increased by miR-21-deficient Treg cells when they were treated with Stattic as determined by both intracellular staining (**Figure 4F**) and ELSIA (**Figure 4G**).

**Figure 5:**
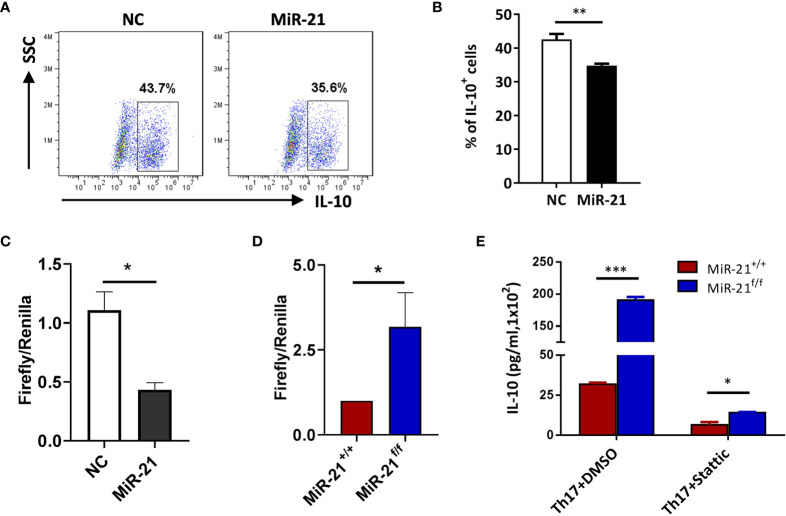
IL-10 is a direct target of miR-21 in Treg cells. **(A, B)** CD4^+^CD25^+^ Treg cells were isolated from the spleen of C57BL/6 mice using EasySep Mouse CD4^+^CD25^+^ Treg Cell Isolation Kit and cultured with α-CD3 plus α-CD28 and IL-2 for 48 h before control adenovirus (NC) or adenovirus over-expressing miR-21 (miR-21) were added. After culture for another 48 h, cells were treated with Th17 priming cytokines for 72 h. Cells were then fixed, permeabilized, stained with anti–IL-10 and examined by flow cytometry **(A)**. The quantification of the proportion of IL-10^+^ cells was shown in **(B)**. Cells shown in **(A)** were gated on transduced cells. **(C, D)** CD4^+^CD8^-^YFP^+^ cells were flow sorted from the spleen of Foxp3^Cre-YFP^miR-21^+/+^ (MiR-21^+/+^) and Foxp3^Cre-YFP^miR-21^f/f^ (MiR-21^f/f^) mice (n=5). MiR-21 agomir was electroporated into CD4^+^CD8^-^YFP^+^ cells from Foxp3^Cre-YFP^miR-21^+/+^ mice together with mouse IL-10 3’UTR reporter construct **(C)**. Alternatively, mouse IL-10 3’UTR reporter construct was electroporated into CD4^+^CD8^-^YFP^+^ cells from Foxp3^Cre-YFP^miR-21^+/+^ and Foxp3^Cre-YFP^miR-21^f/f^ mice **(D)**. Cells were then cultured for 24 h and luciferase activity was examined using a dual-luciferase reporter assay system. **(E)** CD4^+^CD8^-^YFP^+^ cells were flow sorted from the spleen of Foxp3^Cre-YFP^miR-21^+/+^ (MiR-21^+/+^) and Foxp3^Cre-YFP^miR-21^f/f^ (MiR-21^f/f^) mice (n=5). Cells were pre-treated with either DMSO or Stat3 inhibitor (Stattic) for 1h and then cultured with α-CD3 plus α-CD28 in the presence of Th17 priming cytokines for 72 h. Culture supernatants were collected and the protein level of IL-10 was determined by ELISA. Data are representative of three separate experiments. *p < 0.05, **p < 0.01, ***p < 0.001.

In **Figure 2** we have shown that miR-21-deficient Treg cells produce significant more IL-10, this indicates that miR-21 in Treg cells may down-regulate IL-10 expression. We confirmed this hypothesis by showing that adenoviral delivery of miR-21 into Treg cells is able to reduce the proportion of IL-10^+^ cells (**Figures 5A, B**). To determine how miR-21 in Treg cells suppresses IL-10 expression, reporter plasmid of mouse IL-10 3’UTR together with control agomir or miR-21 agomir were electroporated into WT Tregs, and then luciferase activity was measured. Our results showed that luciferase activity was significantly lower when miR-21 level was increased in Treg cells (**Figure 5C**). Furthermore, reporter plasmid of mouse IL-10 3’UTR was electroporated into both WT and miR-21-deficient Treg cells, and then luciferase activity was measured. We found that luciferase activity was significantly higher in miR-21-deficient Treg cells (**Figure 5D**). Since it has previously been shown that the 3’UTR of IL-10 contains potential miR-21 binding site and mutagenesis analysis confirmed that IL-10 is a valid target of miR-21 (20), we believe that miR-21 in Treg cells may suppress IL-10 expression through direct targeting of IL-10.

It has been reported that, although Stat3 is a critical transcription factor for Th17 differentiation, Treg cells suppresses Th17-specific immune response upon activation of Stat3 (32, 33). Since we have shown that Stat3 activity is increased in miR-21-deficient Treg cells, it is possible that those Treg cells may display improved function in suppressing Th17 response. IL-10 is implicated in Stat3-dependent promotion of Treg cell function. However, we have shown in **Figure 4** that miR-21-deficient Treg cells actually produce significant more IL-10. We further demonstrated that, although Stat3 inhibition leads to the down-regulation of IL-10, miR-21-deficient Treg cells still produce significant more IL-10 (**Figure 5E**). We next examined the expression of CCR6, Prf1, Gzmb, and Ebi3, all of which are also implicated in Stat3-dependent promotion of Treg cell function. Our results showed that none of them displayed significant difference between WT and miR-21-deficient Treg cells (**Figure S8**).

### MiR-21 in Tregs Is Largely Dispensable for the Development of Autoimmune Diseases

Since Treg cells that are defective in inhibiting effector T cells *in vivo* may display normal *in vitro* suppressive activity, we next examined whether miR-21 affects Treg function *in vivo* using three mouse models of autoimmune disease, all of which are closely related to Th17-mediated inflammatory immune pathogenesis.

**Figure 6:**
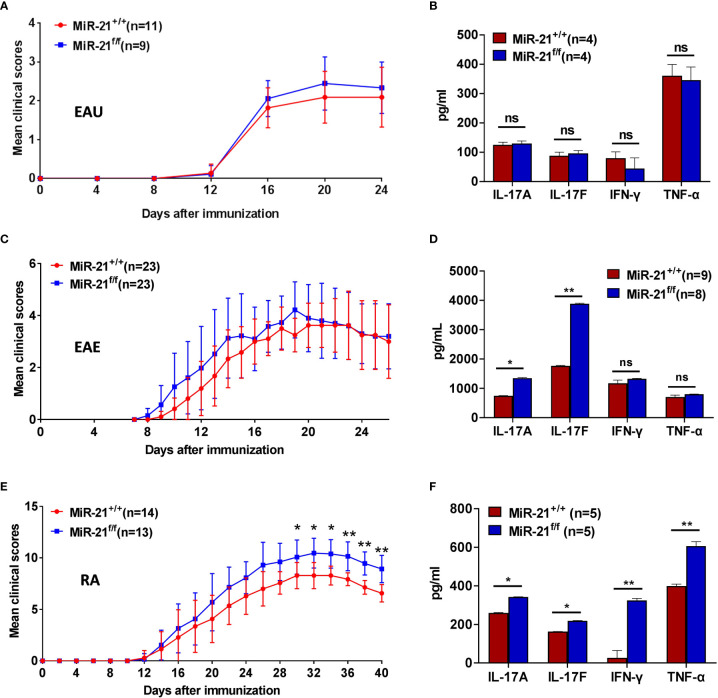
MiR-21 in Tregs is largely dispensable for the development of autoimmune diseases. Mouse models of EAU **(A, B)**, EAE **(C, D)**, and RA **(E, F)** were established in both Foxp3^Cre-YFP^miR-21^+/+^ (MiR-21^+/+^) and Foxp3^Cre-YFP^miR-21^f/f^ (MiR-21^f/f^) mice as described in the materials & methods. The mean clinical scores of EAU **(A)**, EAE **(C)**, and RA **(E)** were determined as described in the materials & methods. Results were combined from two independent experiments. Mice were sacrificed at the 24th (for EAU), 26th (for EAE) or 40th (for RA) day after immunization. Tissue extracts were prepared from eye **(B)**, brain **(D)** and paw **(F)** and concentrations of IL-17A, IL-17F, IFN-γ, and TNF-α in the tissue extracts were determined by ELISA. For **(B, D, F)**, data are representative of two independent experiments. EAU, experimental autoimmune uveoretinitis; EAE, experimental autoimmune encephalomyelitis; RA, rheumatoid arthritis; ns, no significance. *p < 0.05, **p < 0.01.

Experimental Autoimmune Uveitis (EAU) is the animal model of human uveitis that most closely resembles sympathetic ophthalmia. IRBP-induced EAU model was established in both WT and Foxp3^Cre-YFP^miR-21^f/f^ mice. Fundoscopic exam was used to assess the degree of fundus inflammation at different time-points after immunization, and we found no significant difference between WT and Foxp3^Cre-YFP^miR-21^f/f^ mice (**Figure 6A**). In consistency with the phenotypical profiles, there is no significant difference in the concentration of IL-17A, IL-17F, IFN-γ and TNF-α in the eye between WT and Foxp3^Cre-YFP^miR-21^f/f^ mice (**Figure 6B**).

Experimental autoimmune encephalomyelitis (EAE) is the most commonly used rodent model of multiple sclerosis (MS). MOG peptide-induced EAE model was established in both WT and Foxp3^Cre-YFP^miR-21^f/f^ mice. There is no significant difference in clinical scores except that Foxp3^Cre-YFP^miR-21^f/f^ mice exhibit a slightly increased severity during the induction phase (**Figure 6C**). When the concentration of inflammatory cytokines in the brain was examined, we found no significant difference in the concentration of IFN-γ and TNF-α between WT and Foxp3^Cre-YFP^miR-21^f/f^ mice. However, the concentration of IL-17 was increased in Foxp3^Cre-YFP^miR-21^f/f^ mice (**Figure 6D**).

The collagen-induced arthritis (CIA) mouse model is the most commonly studied autoimmune model of rheumatoid arthritis. Collagen II-induced CIA model was established in both WT and Foxp3^Cre-YFP^miR-21^f/f^ mice. Mice were examined physically every other day and four-point arthritis indexes were assessed. As shown in **Figure 6E**, Foxp3^Cre-YFP^miR-21^f/f^ mice exhibit a slightly increased but significant disease severity than WT mice. In consistency with the phenotypical profiles, the concentrations of inflammatory cytokines including IL-17A, IL-17F, IFN-γ and TNF-α were all significantly increased in the paw of Foxp3^Cre-YFP^miR-21^f/f^ mice (**Figure 6F**).

Taking together, these data indicate that although Foxp3^Cre-YFP^miR-21^f/f^ mice exhibit a slightly increased RA severity than WT mice, miR-21 in Treg cells is largely dispensable for the development of autoimmune disease.

### MiR-21-Deficient Treg Cells That Have Experienced Inflammatory Environment *In Vivo* Produce Significant More IL-17 and IL-10

**Figure 7:**
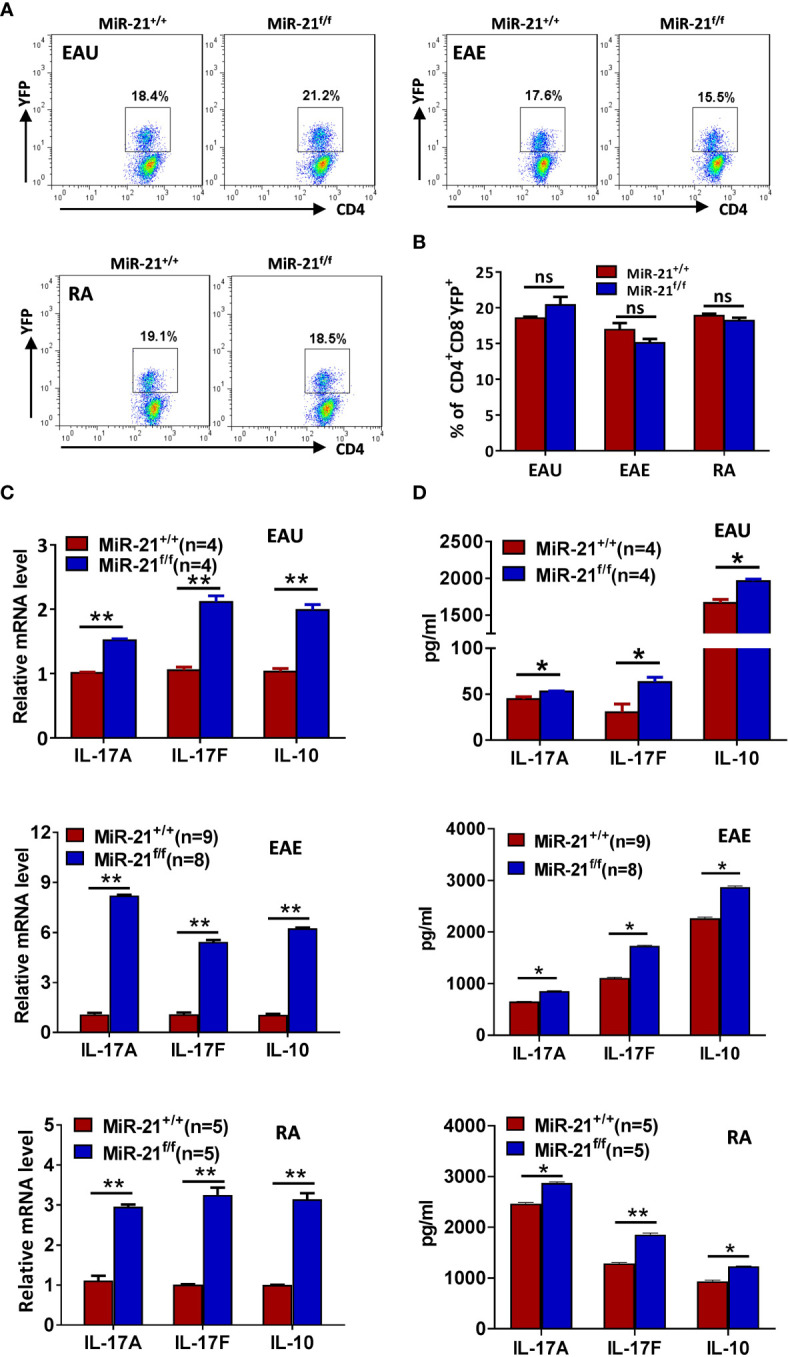
MiR-21-deficient Treg cells that have experienced inflammatory environment *in vivo* produce significant more IL-17 and IL-10. Mouse models of EAU, EAE, and RA were established and mice were sacrificed as in **Figure 7**. **(A, B)** The percentage of CD4^+^CD8^-^YFP^+^ Treg cells in the CLN (for EAU and EAE) and ILN (for RA) were determined by flow cytometry **(A)**. Cells shown were gated on CD4^+^CD8^-^ cells. The quantification of the percentage of CD4^+^CD8^-^YFP^+^ Treg cells was shown in **(B). (C, D)** CD4^+^CD8^-^YFP^+^ cells were flow sorted from the CLN (for EAU and EAE) and ILN (for RA). Total RNA was extracted and the mRNA expression of IL-17A, IL-17F, and IL-10 was determined by quantitative RT-PCR **(C)**. In addition, sorted CD4^+^CD8^-^YFP^+^ cells were cultured with α-CD3 plus α-CD28 and IL-2 for 48 h. Culture supernatants were then collected and the protein level of IL-17A, IL-17F, and IL-10 was determined by ELISA **(D)**. Data are representative of two independent experiments. CLN, cervical lymph node; ILN, inguinal lymph node; ns, no significance. *p < 0.05, **p < 0.01.

Since we have shown that IL-17 and IL-10 production was increased by miR-21-deficient Treg cells upon treatment *in vitro* with pathogenic Th17-priming cytokines, we then examined whether they also produce more IL-17 and IL-10 after experiencing inflammatory environment *in vivo*. In all three autoimmune disease models studied, the proportion of CD4^+^CD8^-^YFP^+^ cells in the draining lymph node was comparable between WT and Foxp3^Cre-YFP^miR-21^f/f^ mice (**Figures 7A, B**). In consistency with the *in vitro* results from **Figure 4**, we found that the mRNA (**Figure 7C**) and protein levels (**Figure 7D**) of IL-17A, IL-17F, and IL-10 were significant increased by Treg cells from Foxp3^Cre-YFP^miR-21^f/f^ mice in all three autoimmune disease models studied.

## Discussion

The expression of miR-21 is enhanced in multiple solid tumors and lymphomas where it regulates transformation by limiting the expression of various tumor suppressor genes (34). Outside of tumors, miR-21 is also highly expressed in Th17 and Treg cells (17). While studies have shown that miR-21 promotes Th17 differentiation and glucose metabolism of pathogenic Th17 cells, our current study suggests that miR-21 in Treg cells suppresses the expression of IL-17 and IL-10 under inflammatory condition but is largely dispensable for the development of autoimmune disease.

Since Stat3 is a direct target of miR-21 (35, 36) and plays a pivotal role in determining Th17 differentiation (37, 38), it is not surprising that we found miR-21 suppresses IL-17 production by Treg cells through down-regulating Stat3 activity. However, studies have also found that miR-21 in naïve CD4^+^ T cells promotes Th17 differentiation through the down-regulation of Smad7 and Peli1, as miR-21-deficient CD4^+^ T cells display impaired Th17 differentiation (17, 23). We hypothesize that whether miR-21 suppresses or promotes IL-17 production depends on the level of miR-21, and excessive miR-21 in conventional CD4^+^ T cells may also suppress Th17 differentiation. Our un-published data partially confirmed this hypothesis by showing that TNF-α in combination with pathogenic Th17-priming condition will significantly increase miR-21 expression and down-regulate Th17 differentiation. Although further study is needed, this preliminary data indicate that miR-21 may also suppress Th17 differentiation when under strong miR-21-inducing condition.

It has been generally recognized that the same transcription factor integrate environmental cues that guide a particular immune response type and facilitate Treg cell ability to suppress the corresponding type of the immune response (32, 39, 40). Although Stat3 is a cytokine-activated essential regulator in Th17 development, it has been shown that phosphor-Stat3 associates with Foxp3 in Tregs and Stat3-deficient Tregs are selectively impaired in their ability to control Th17 responses (32). However, our current study suggests that Stat3 activation in Tregs under inflammatory condition must be tightly controlled, as excessive Stat3 activation in Tregs will lead to the up-regulation of IL-17. Since miR-21 expression is increased in Tregs upon treatment with pathogenic Th17-priming condition, we hypothesize that miR-21 may fine-tune the function of Stat3 by acting as a negative regulator of Stat3.

The plasticity and functional adaptability of Tregs under an inflammatory microenvironment has been demonstrated in autoimmunity (9). Inflammatory condition can cause the loss of Foxp3 expression and the generation of exTregs that adopt a phenotype that is more characteristic of effector CD4^+^ T cells (7). Foxp3^+^ cells are comprised of Foxp3-stable CD25^hi^ and Foxp3-unstable CD25^lo^ populations, the former of which is composed of bona fide Treg cells with sustained Foxp3 expression. Th17 cells originating from CD25^lo^Foxp3^+^ T cells have a key role in the pathogenesis of autoimmune diseases (8). Alternatively, a faction of Tregs may display increased plasticity toward effector cells but retained Foxp3 expression and suppressive function (10). In the current study, we showed that miR-21-deficient and sufficient Tregs exhibit similar degree of conversion to exTregs when cultured *in vitro* under pathogenic Th17-priming condition. Furthermore, we demonstrated that miR-21-deficient Tregs, whether lost or retained Foxp3 expression, produced significant more IL-17. A fraction of Treg cells may lose YFP expression and convert to exTregs during the development of autoimmune disease. In our *in vivo* studies we only examined the percentage of YFP^+^ cells in the draining lymph nodes and the production of IL-17 and IL-10 by them, whether miR-21 regulates the stability of Tregs *in vivo* warrants further investigation. We are currently working on this using double transgenic mouse model that permits Foxp3 lineage tracing.

The pathological importance of Foxp3 instability in the generation of pathogenic Th17 cells in autoimmunity has been well established. However, although our study showed that miR-21-deficient Tregs produce significant more IL-17 compared with miR-21-sufficient Tregs, miR-21 in Tregs is dispensable for the development of EAU and EAE, and miR-21-deficiency in Tregs only slightly increased the disease severity in RA mouse model. The development of inflammatory autoimmune disease often depends on the balance between Th1/Th17 cells and Tregs. It is likely that increased IL-10 expression by miR-21-deficient Tregs may offset the pathogenic effects mediated by increased Th17 response (**Figure S9**). However, when under certain inflammatory condition, such as that in the RA model, miR-21-deficiency in Tregs may cause the imbalance between Th17 cells and Tregs. There is strong evidence showing that plastic Foxp3^+^ T cells contribute to the pathogenesis of RA. When under arthritic conditions, Treg cells lose Foxp3 expression and undergo trans-differentiation into Th17 cells. These IL-17-expressing exTregs accumulated in inflamed joints and were even more potent osteoclastogenic T cells than were naive CD4^+^ T cell–derived Th17 cells (8). It is worth noting that increased Stat3 in Tregs may promote the expression of other genes implicated in Treg suppressive function. However, we examined the expression of several key genes (CCR6, Ebi3, Gzmb and Prf1) that have been implicated in Stat3-dependent promotion of Treg function and found none of them displayed significant difference between WT and miR-21-deficient Treg cells.

In conclusion, our study demonstrated that miR-21 in Tregs regulates diametrically opposed biological Treg functions and whether it regulates the development of autoimmune disease depends on the specific inflammatory environment of different types of disease.

## Data Availability Statement

The original contributions presented in the study are included in the article/**Supplementary Material**. Further inquiries can be directed to the corresponding authors.

## Ethics Statement

The animal study was reviewed and approved by Shandong Institute of Ophthalmology, Shandong First Medical University.

## Author Contributions

QR designed research. JS, RL, XH, JB, and WZ performed research. RL contributed reagents/analytic tools. JS, WS, and QR analyzed data. JS and QR wrote the paper. All authors contributed to the article and approved the submitted version.

## Funding

This study was supported by Qingdao Municipal Science and Technology Bureau (21-1-4-rkjk-11-nsh).

## Conflict of Interest

The authors declare that the research was conducted in the absence of any commercial or financial relationships that could be construed as a potential conflict of interest.

## Publisher’s Note

All claims expressed in this article are solely those of the authors and do not necessarily represent those of their affiliated organizations, or those of the publisher, the editors and the reviewers. Any product that may be evaluated in this article, or claim that may be made by its manufacturer, is not guaranteed or endorsed by the publisher.
