# Supplementary material for: MicroRNA-21 Regulates Diametrically Opposed Biological Functions of Regulatory T Cells
Source: Front Immunol. 2021 Nov 11;12:766757. doi: 10.3389/fimmu.2021.766757 (PMC8632542; doi:10.3389/fimmu.2021.766757)
Supplement: Supplementary file 1 [file DataSheet_1.pdf]

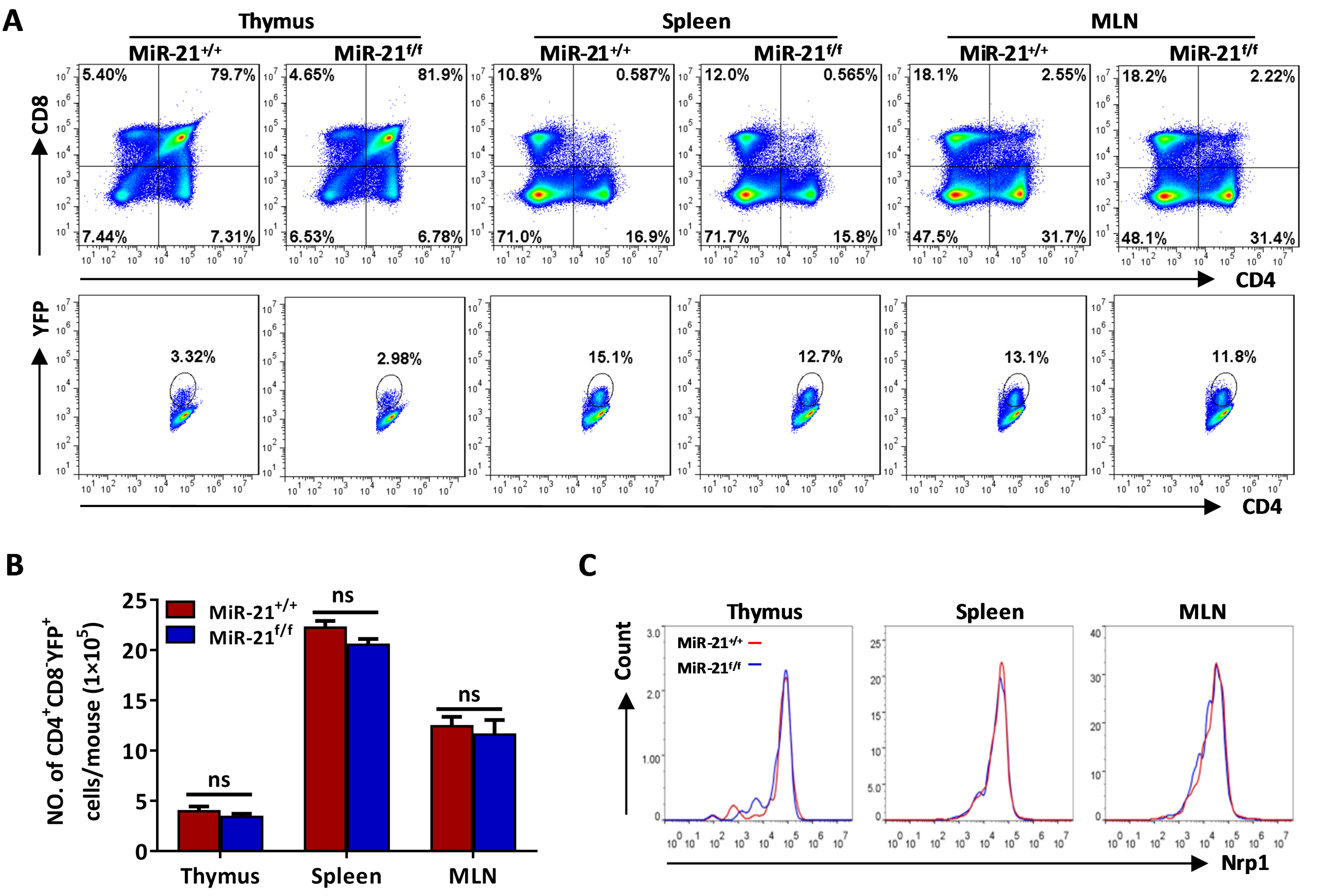

Figure S1. Thymic and peripheral T cell development were normal in Foxp3$^{Cre\text{-}YFP}$miR-21$^{f/f}$ mice .

Total cells from the thymus, spleen and MLN of Foxp3$^{Cre\text{-}YFP}$miR-21$^{+/+}$ (MiR-21$^{+/+}$) and Foxp3$^{Cre\text{-}YFP}$miR-21$^{f/f}$ (MiR-21$^{f/f}$) mice (n=4) were isolated and stained with fluorescent-labeled antibodies against CD4 and CD8, and then analyzed by flow cytometry **(A)**. The number of Tregs (CD4$^+$CD8$^-$YFP$^+$) in each organ was calculated **(B)**. Alternatively, cells were stained with fluorescent-labeled antibodies against CD4, CD8, Nrp1, and analyzed by flow cytometry **(C)**. Cells shown in (C) were gated on CD4$^+$CD8$^-$YFP$^+$ cells. Results are representative of two independent experiments. MLN, mesenteric lymph node; ns, no significance.

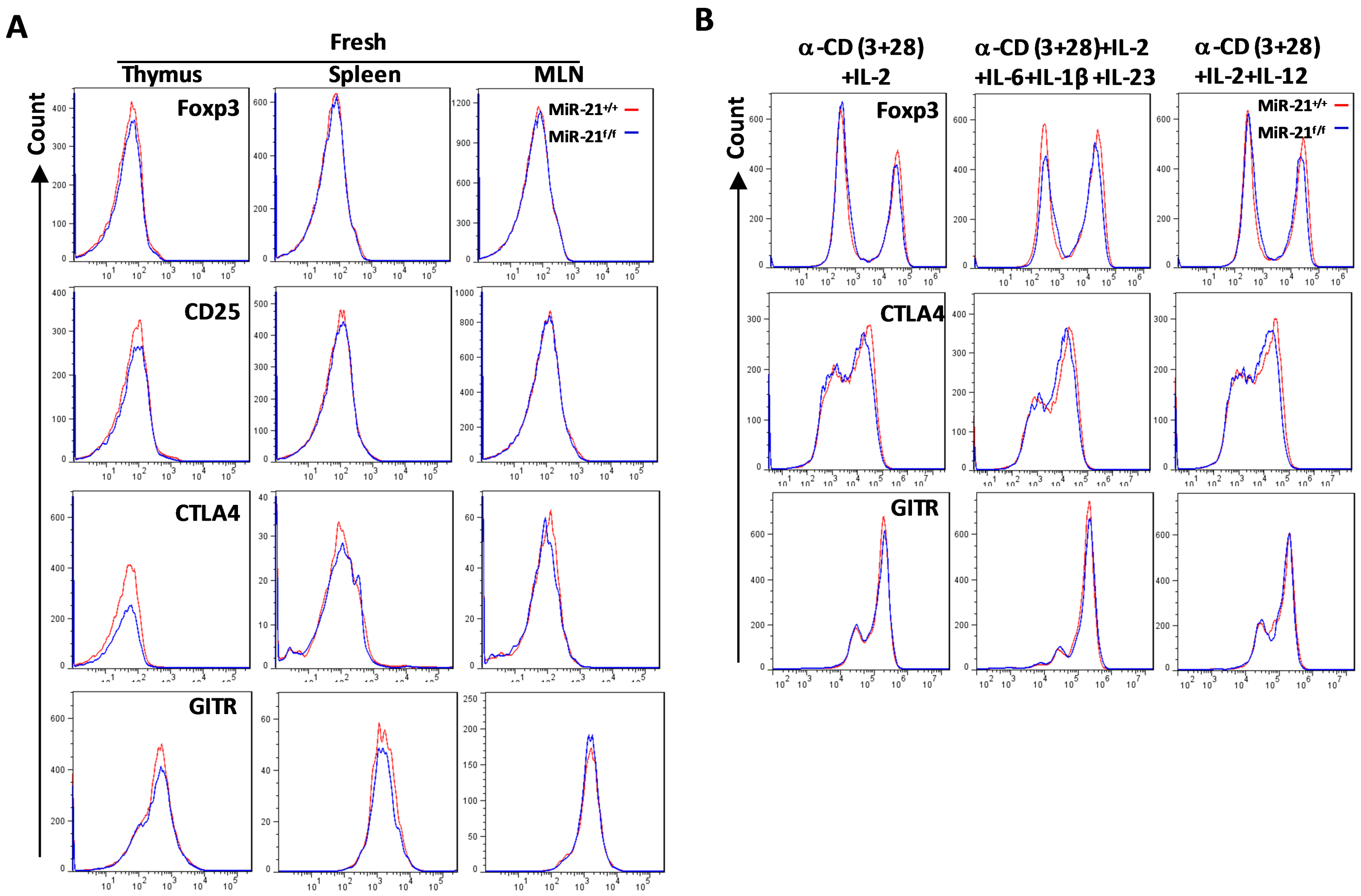

Figure S2. Treg cells lacking miR-21 display normal phenotype.

Total cells were isolated from the spleen of Foxp3$^{Cre\text{-}YFP}$miR-21$^{+/+}$ (MiR-21$^{+/+}$) and Foxp3$^{Cre\text{-}YFP}$miR-21$^{f/f}$ (MiR-21$^{f/f}$) mice (n=5). CD4^{+}CD8^{-}YFP^{+} cells were flow sorted and either untreated **(A)** or treated *in vitro* under the indicated conditions for 72 h **(B)**. Cells were then stained with fluorescent-labeled antibodies against Foxp3, CD25, CTLA4, or GITR, and analyzed by flow cytometry. Results are representative of three independent experiments. MLN, mesenteric lymph node.

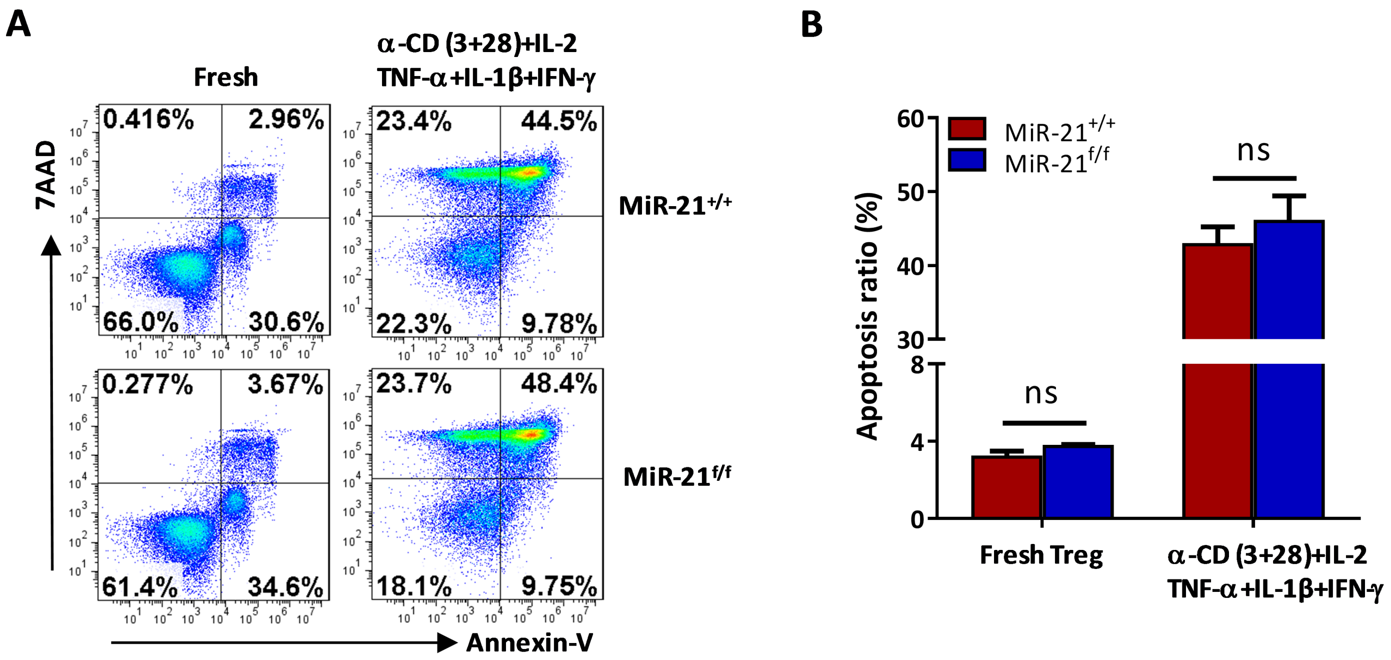

Figure S3. MiR-21 deficiency doesn't affect the apoptosis of Treg cells.
$CD4^+CD8^-YFP^+$ cells were flow sorted from the spleen of Foxp3$^{Cre\text{-}YFP}$miR-21$^{+/+}$ (MiR-21$^{+/+}$) and Foxp3$^{Cre\text{-}YFP}$miR-21$^{f/f}$ (MiR-21$^{f/f}$) mice (n=3). Cells were then untreated (fresh) or treated with α-CD3 plus α-CD28 in the presence of IL-2, TNF-α, IL-1β and IFN-γ for 96 h. Cells were stained with APC-conjugated Annexin-V and 7AAD, and analyzed by flow cytometry **(A)**. The proportion of apoptotic cells (Annexin V$^+$7AAD$^+$) was shown in **(B)**. Results are representative of two independent experiments. ns, no significance.

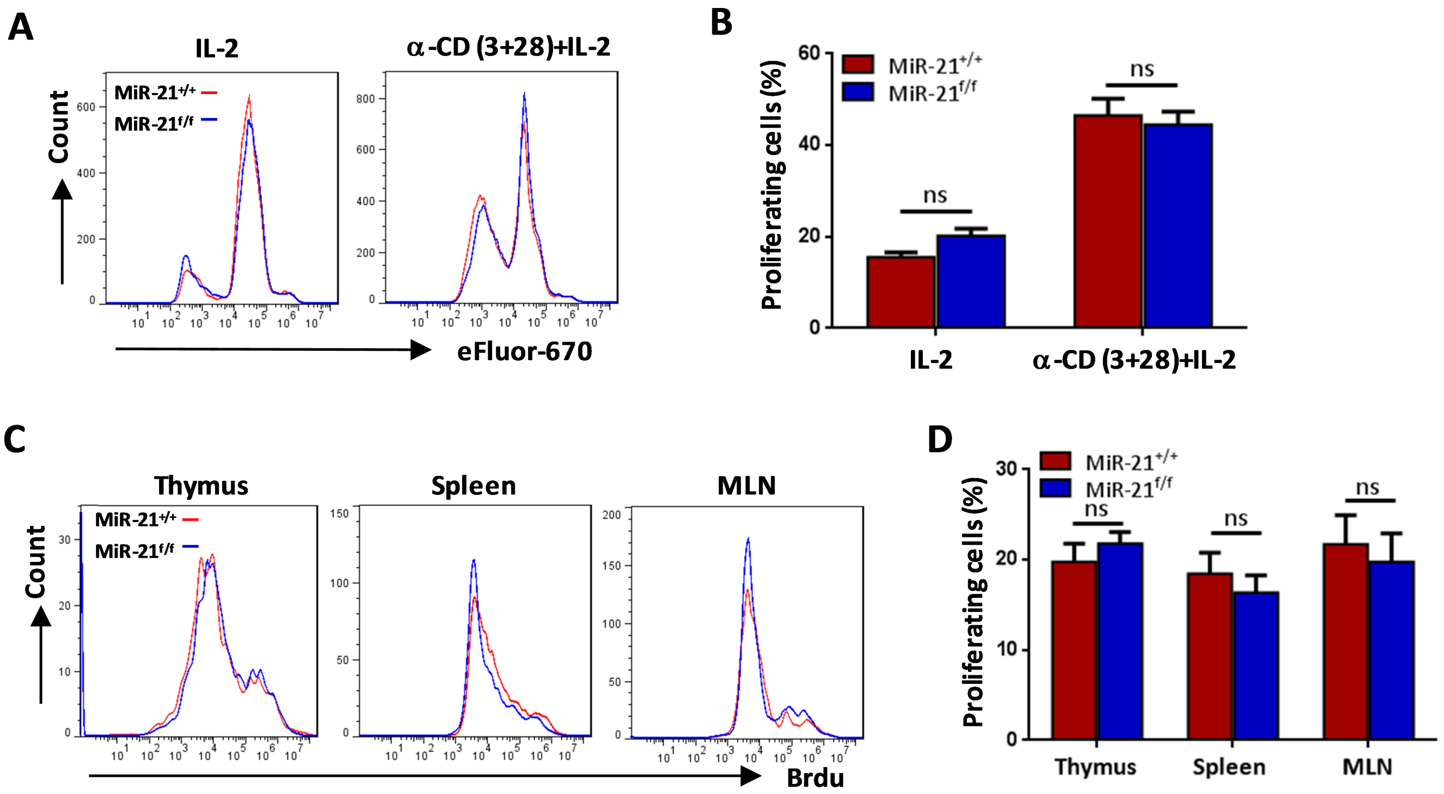

Figure S4. MiR-21 deficiency doesn't affect the proliferation of Treg cells.
(A & B) Flow sorted splenic $CD4^+CD8^-YFP^+$ cells were labeled with eFluor-670 and then treated with 100 IU/ml IL-2 in the absence or presence of α-CD3 plus α-CD28. After 72 h, cell proliferation was examined by flow cytometry **(A)**. Quantification of the percentage of proliferating Treg cells was shown in **(B)**. Proliferating cells were defined as those with diluted eFluor-670. **(C & D)** 6 to 8-week-old $Foxp3^{Cre\text{-}YFP}$miR-$21^{+/+}$ (MiR-$21^{+/+}$) and miR-$21^{f/f}$ mice (MiR-$21^{f/f}$) (n=4) were intraperitoneally injected with BrdU as described in the materials & methods. 12 h after the last treatment, mice were sacrificed and total cells were isolated from thymus, spleen, and MLN. Cell surface staining was first performed with fluorescent-labeled antibodies against CD4 and CD8 followed by intracellular staining with fluorescent-labeled antibody against BrdU. Stained cells were analyzed by flow cytometry **(C)**. Quantification of the percentage of proliferating Treg cells ($CD4^+CD8^-YFP^+BrdU^+$) was shown in **(D)**. Cells shown in (C) were gated on $CD4^+CD8^-YFP^+$ cells. Data are representative (A & C) or combination (B & D) of two independent experiments. MLN, mesenteric lymph node; ns, no significance.

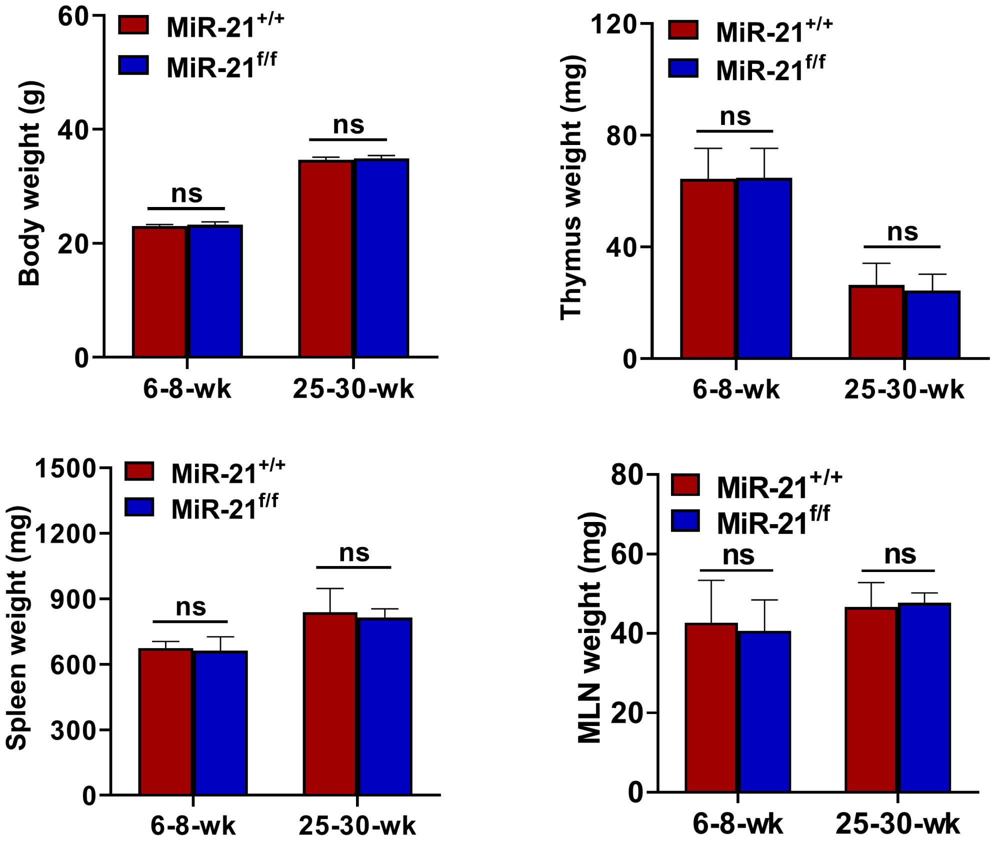

Figure S5. No splenomegaly or lymphadenopathy was observed in Foxp3$^{Cre\text{-}YFP}$miR-21$^{f/f}$ mice.

Foxp3$^{Cre\text{-}YFP}$miR-21$^{+/+}$ (MiR-21$^{+/+}$) and Foxp3$^{Cre\text{-}YFP}$miR-21$^{f/f}$ (MiR-21$^{f/f}$) mice (n=10) at 6-8-week and 25-30-week of age were sacrificed and the weight of body, thymus, spleen and MLN was determined. MLN, mesenteric lymph node; ns, no significance.

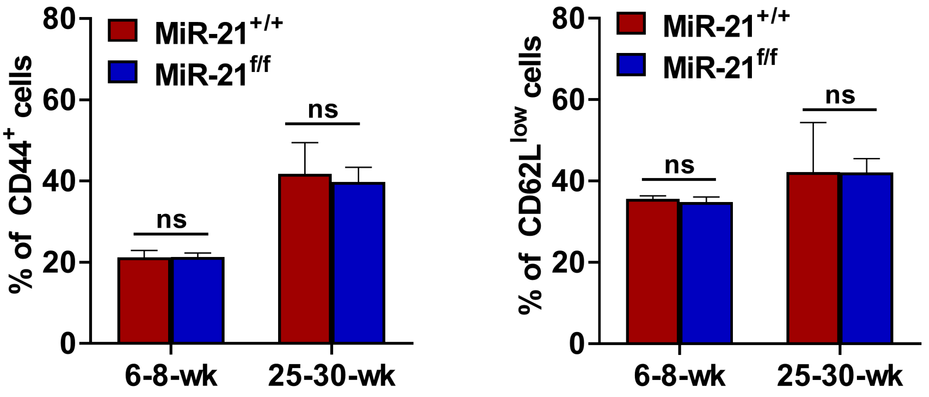

Figure S6. Fractions of naïve and memory T cells among $CD4^{+}CD8^{-}YFP^{-}$ cells were comparable between WT and $Foxp3^{Cre\text{-}YFP}miR\text{-}21^{f/f}$ mice.

Total cells were isolated from the spleen of $Foxp3^{Cre\text{-}YFP}miR\text{-}21^{+/+}$ ($MiR\text{-}21^{+/+}$) and $Foxp3^{Cre\text{-}YFP}miR\text{-}21^{f/f}$ ($MiR\text{-}21^{f/f}$) mice (n=10) at 6-8-week and 25-30-week of age. Cells were then stained with fluorescent-labeled antibodies against CD4, CD8, CD44, CD62L, and analyzed by flow cytometry. ns, no significance.

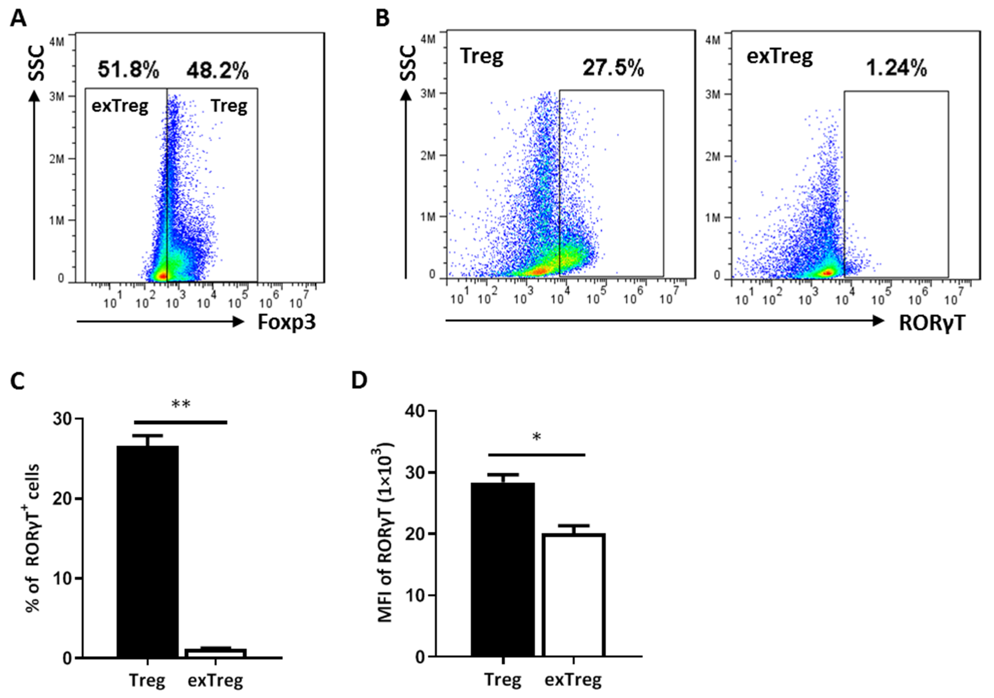

Figure S7.“exTregs” generated *in vitro* exhibit reduced RORγT expression. CD4$^+$CD8$^-$YFP$^+$ cells were flow sorted from the spleen of Foxp3$^{Cre\text{-}YFP}$miR-21$^{+/+}$ mice (n=3). Cells were stimulated with anti-CD3 plus anti-CD28 in the presence of Th17 priming cytokines. After 72 h, cells were treated with PMA plus ionomycin in the presence of GolgiStop for 4 h. Cells were then fixed, permeabilized, stained with fluorochrome-conjugated antibodies against Foxp3 and RORγT, and examined by flow cytometry. The proportions of Foxp3$^+$ (Treg) and Foxp3$^-$ (exTreg) cells among total live cells were shown in **(A)**. In addition, the proportions of RORγT$^+$ cells among Foxp3$^+$ (Treg) and Foxp3$^-$ (exTreg) cells were also examined by flow cytometry **(B)** and the quantification of the percentage of RORγT$^+$ cells **(C)** and the mean fluorescence intensity (MFI) of RORγT **(D)** were shown. Data are representative of two separate experiments. *$p<0.05$, **$p<0.01$.

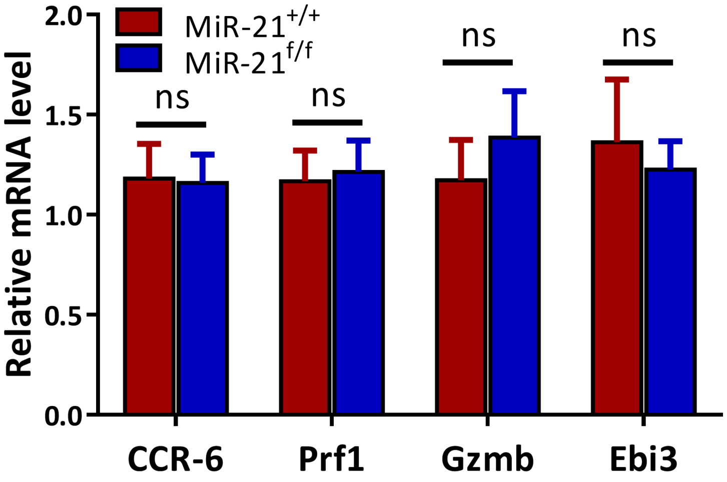

Figure S8. The expression of CCR6, Prf1, Gzmb, and Ebi3 by Treg cells was comparable between WT and Foxp3$^{Cre\text{-}YFP}$miR-21$^{f/f}$ mice.

CD4$^+$CD8$^-$YFP$^+$ cells were flow sorted from the spleen of Foxp3$^{Cre\text{-}YFP}$miR-21$^{+/+}$ (MiR-21$^{+/+}$) and Foxp3$^{Cre\text{-}YFP}$miR-21$^{f/f}$ (MiR-21$^{f/f}$) mice (n=5). Cells were then cultured with anti-CD3 plus anti-CD28 in the presence of Th17-priming cytokines. After 48 h, total RNA was extracted, and the mRNA expression of CCR-6, Prf1, Gzmb, and Ebi3 was determined by quantitative RT-PCR. Data are representative of two separate experiments. ns, no significance.

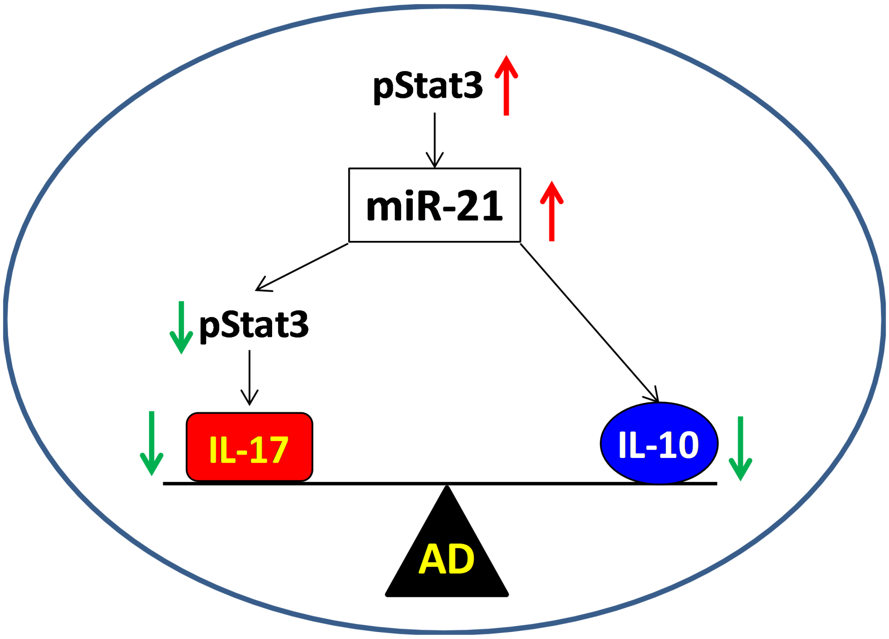

Figure S9. Schematic diagram showing that miR-21 in Treg cells regulates diametrically opposed biological functions.

Level of phosphor-Stat3 (pStat3) increased when Tregs were under pathogenic Th17-priming condition. pStat3 promotes the expression of miR-21, which is a direct target of Stat3. Increased miR-21 level in Tregs will then regulate diametrically opposed biological functions: In one hand, miR-21 suppresses Stat3 activity in a negative feedback manner, since Stat3 is also a direct target of miR-21. This will in turn dampen inflammatory Th17 response because Stat3 is an important regulator of Th17 differentiation. On the other hand, since anti-inflammatory cytokine IL-10 is a direct target of miR-21, increased miR-21 level will lead to the down-regulation of IL-10. Overall, miR-21 in Tregs is largely dispensable for the development of autoimmune disease (AD) because increased IL-10 expression by miR-21-deficient Tregs may offset the pathogenic effects mediated by increased Th17 response.
